# A Universal Plasticizer Alkylsulfonic Phenyl Ester Activates Pregnane X Receptor to Increase Intestinal Cholesterol Uptake

**DOI:** 10.3390/ijms27188189

**Published:** 2026-09-15

**Authors:** Nnamdi Okore, Wangeci Kariuki, Conner Brown, Michael A. Moxley, Naara Ramirez, Yipeng Sui

**Affiliations:** 1 Department of Biology, University of Nebraska at Kearney, Kearney, NE 68849, USA; okoren@lopers.unk.edu (N.O.); stephbah9@gmail.com (W.K.); conner.nehosa@gmail.com (C.B.); naararamirez6@gmail.com (N.R.); 2 Department of Chemistry, University of Nebraska at Kearney, Kearney, NE 68849, USA; moxleyma@unk.edu

**Keywords:** alkylsulfonic phenyl ester, pregnane X receptor, cholesterol uptake, dyslipidemia, cardiovascular disease, endocrine disrupting chemical

## Abstract

Alkylsulfonic phenyl ester (ASE) is an alternate non-phthalate plasticizer used in PVC and polyurethane manufacture, such as for medical tubing and toys. Plastic-associated endocrine disrupting chemicals have been associated with increased cardiovascular disease risks and have been established as the agonists of a xenobiotic sensor, pregnane X receptor (PXR), which has atherogenic and dyslipidemic effects. However, it is unknown whether ASE activates PXR to adversely affect cardiovascular system. Our objective is to investigate how ASE impacts lipid profiles and cholesterol uptake via PXR signaling. Human hepatic and intestinal cells were used to test activation of PXR by ASE. The computational docking study and site-directed mutagenesis were used to identify key amino acid residues in PXR that interacted with ASE. The natural PXR inhibitor resveratrol was used to prohibit PXR activities. The male C57BL/6 wildtype mice were fed with ASE to evaluate if ASE could alter plasma lipid levels. Our data suggested that ASE was a specific PXR agonist and increased atherogenic cholesterol levels in mice mediated by PXR. Furthermore, ASE treatment increased cholesterol uptake by human intestinal cells through PXR signaling. Our study elucidates the role of PXR as a mediator of xenobiotic-elicited dyslipidemia and provides evidence for the future risk assessment of ASE on cardiovascular diseases.

## 1. Introduction

Cardiovascular disease (CVD) remains the leading cause of death worldwide [1]. Our chemical environment has changed substantially in the past few decades, and recent large-scale human studies have implicated a novel link between exposure to endocrine-disrupting chemicals (EDCs) and CVD [2,3]. Plastics have become an integral part of modern life, but plastic pollution poses significant environmental and public health concerns [4]. Various plasticizers have been identified as EDCs and have been linked to an increased risk of CVD in humans [5,6]. Nevertheless, the mechanisms underlying the EDC-associated CVD remain poorly understood, limiting our ability to precisely assess the risks associated with exposure to EDCs.

Many plastic-associated EDCs, such as bisphenol A and phthalates, have been identified as potent agonists of a nuclear receptor pregnane X receptor (PXR), which is activated by a wide range of dietary steroids, pharmaceutical agents, and environmental chemicals [7,8,9,10,11]. Beyond its established role in xenobiotic metabolism, PXR has also been implicated in promoting atherosclerosis development in animal models [12,13]. However, the detailed mechanisms responsible for the atherogenic effects of PXR are not completely understood. Our preliminary data suggested that PXR could be activated by a non-phthalate plasticizer, alkylsulfonic phenyl ester (ASE), which is widely used in the production of polyvinyl chloride (PVC) and consumer products and used as a substitute for several phthalates in polymer and non-polymer applications [14].

ASE is manufactured at a rate of 10–50 million pounds per year in the U.S. [15] and is approved for use in PVC food contact articles in the U.S. and Europe [16]. It has been reported that ASE is specifically used in children’s products [15], such as toys and medical tubing [17,18]. Dust ingestion is an important non-dietary exposure route, and the estimated daily dust ingestion exposure rates for children are 0.08–0.86 µg/kg bodyweight. However, these are external exposure estimates and are not measured blood or tissue concentrations, so 0.08–0.86 µg/kg/day should not be interpreted as total human exposure [19]. Infants and children may ingest ASE by mouthing products containing ASE or by the dust contaminated with ASE, while the general population may be exposed to ASE via food ingestion by food contact and packaging materials [16] although dietary and other exposure routes were insufficiently characterized. In Europe, the Scientific Panel of Food Contact Materials, Enzymes, Flavourings and Processing Aids does not allow ASE to be in contact with fatty foods [20]. ASE displayed low systemic toxicity in rats [21]; however, it is unknown whether ASE has adverse impacts on CVD such as dyslipidemia and atherosclerosis. A phthalate plasticizer di(2-ethylhexyl) phthalate (DEHP) has been linked to elevated blood pressure levels and the changes in triglycerides and lipoproteins in an epidemiologically representative sample of the US pediatric population [22]. The extraction rate of ASE from PVC into water is greater than DEHP [16], implying the potential cardiometabolic health risk of ASE exposure. PXR signaling is involved in the regulation of lipid homeostasis; therefore, PXR is an important mediator of EDC influenced disease [8,10,12,23,24,25,26]. However, it is still unknown how ASE activates PXR signaling.

Given the potential association between ASE exposure and CVD, we hypothesized that ASE could promote PXR-mediated intestinal cholesterol uptake, which resulted in increased plasma cholesterol levels. Both human hepatic and intestinal cells were utilized to test how ASE activated PXR. By combining molecular docking study and site-directed mutagenesis, we identified key ASE/PXR interactions. To determine how ASE could alter in vivo plasma lipid profiles via PXR signaling, C57BL/6 wildtype mice were orally fed ASE in the presence of a natural PXR antagonist resveratrol (RES). Furthermore, human intestinal cells were treated with ASE and RES to estimate the impacts of ASE on cholesterol uptake. Our study aims to investigate the potential risks of dyslipidemia associated with ASE exposure and elucidate the underlying mechanisms.

## 2. Results

### 2.1. Assessment of ASE as a Potential PXR Agonist

**Figure 1:**
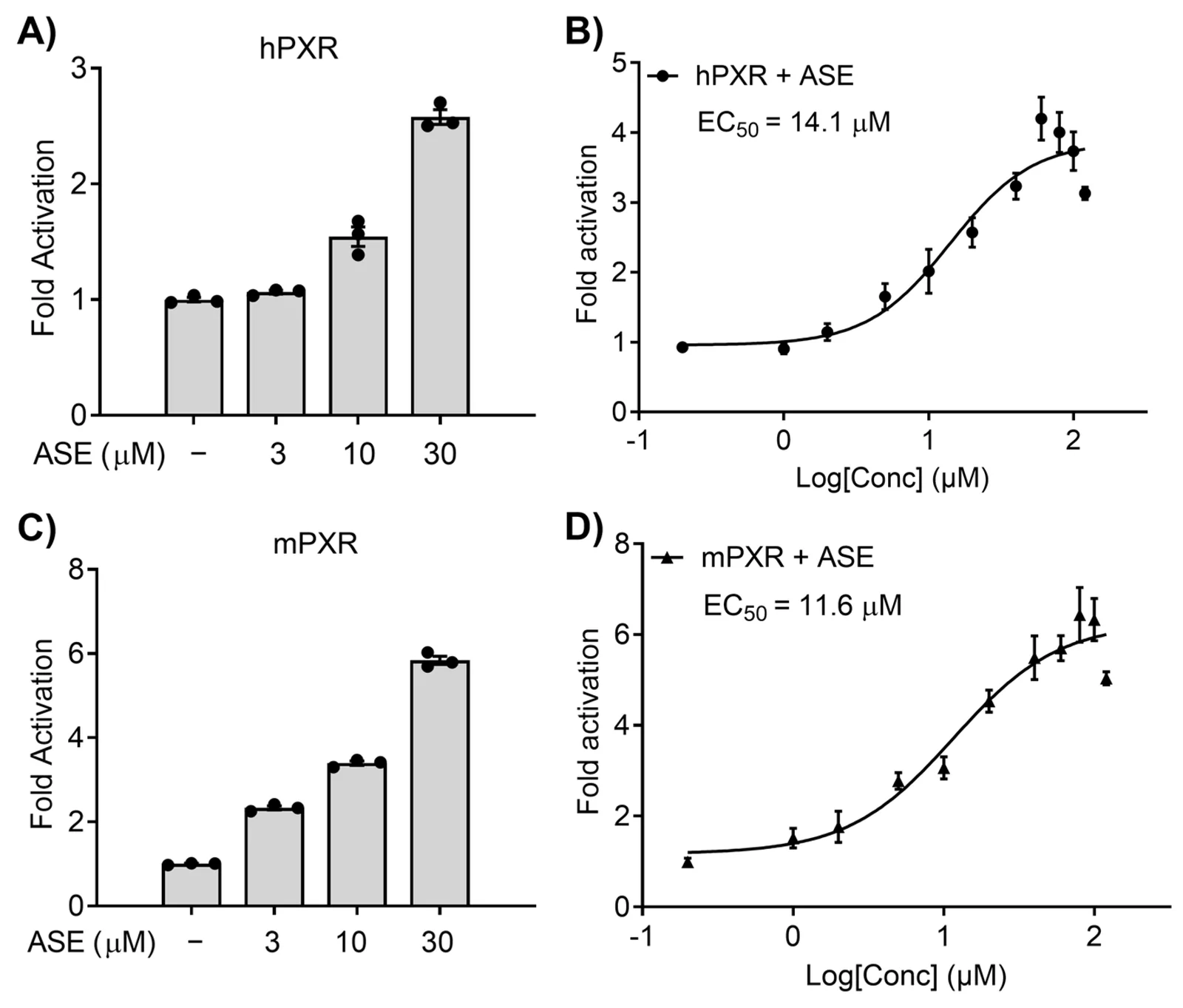
The impacts of ASE on PXR activity in human hepatic HepG2 cells using transfection assay. (**A**,**B**) Full-length hPXR plasmid, hPXR reporter CYP3A4-luc, and β-galactosidase (β-gal) control plasmids were co-transfected. Subsequently, HepG2 cells were treated with ASE (**A**) at the indicated concentrations or (**B**) at doses from 0.2 to 120 µM for 24 h (n = 3). (**C**,**D**) HepG2 cells were transfected with full-length mPXR plasmid, mPXR reporter (CYP3A2)_3_-luc, and β-gal control plasmids, followed by treatment of ASE (**C**) at the indicated concentrations or (**D**) at doses from 0.2 to 120 µM for 24 h (n = 3). Reporter activity was normalized to the β-gal transfection controls and expressed as Relative Light Units (RLUs) per OD_420_ β-gal per minute to facilitate comparisons between plates. Fold activation was calculated relative to vehicle (DMSO) controls. Error bars represent ± SEM.

To evaluate ASE cytotoxicity, both HepG2 and LS180 cells were treated with ASE at 3, 10, 30, and 100 µM. The standard 3-(4,5-dimethylthiazol-2-yl)-2,5-diphenyltetrazolium bromide (MTT) assay suggested that ASE at the indicated doses did not affect cell survival compared with control in either cell line (Supplementary Figure S1). A cell-based transfection assay was used to test the ability of ASE to activate human PXR (hPXR) and mouse PXR (mPXR) in human HepG2 hepatic cells. ASE induced PXR reporter activities in both hPXR and mPXR reporters in a dose-dependent manner (Figure 1A,C). Based on dose response curve analysis, the EC_50_ for ASE activation of hPXR activity was 14.1 μM (Figure 1B), and the EC_50_ for mPXR activity was 11.6 μM (Figure 1D).

**Figure 2:**
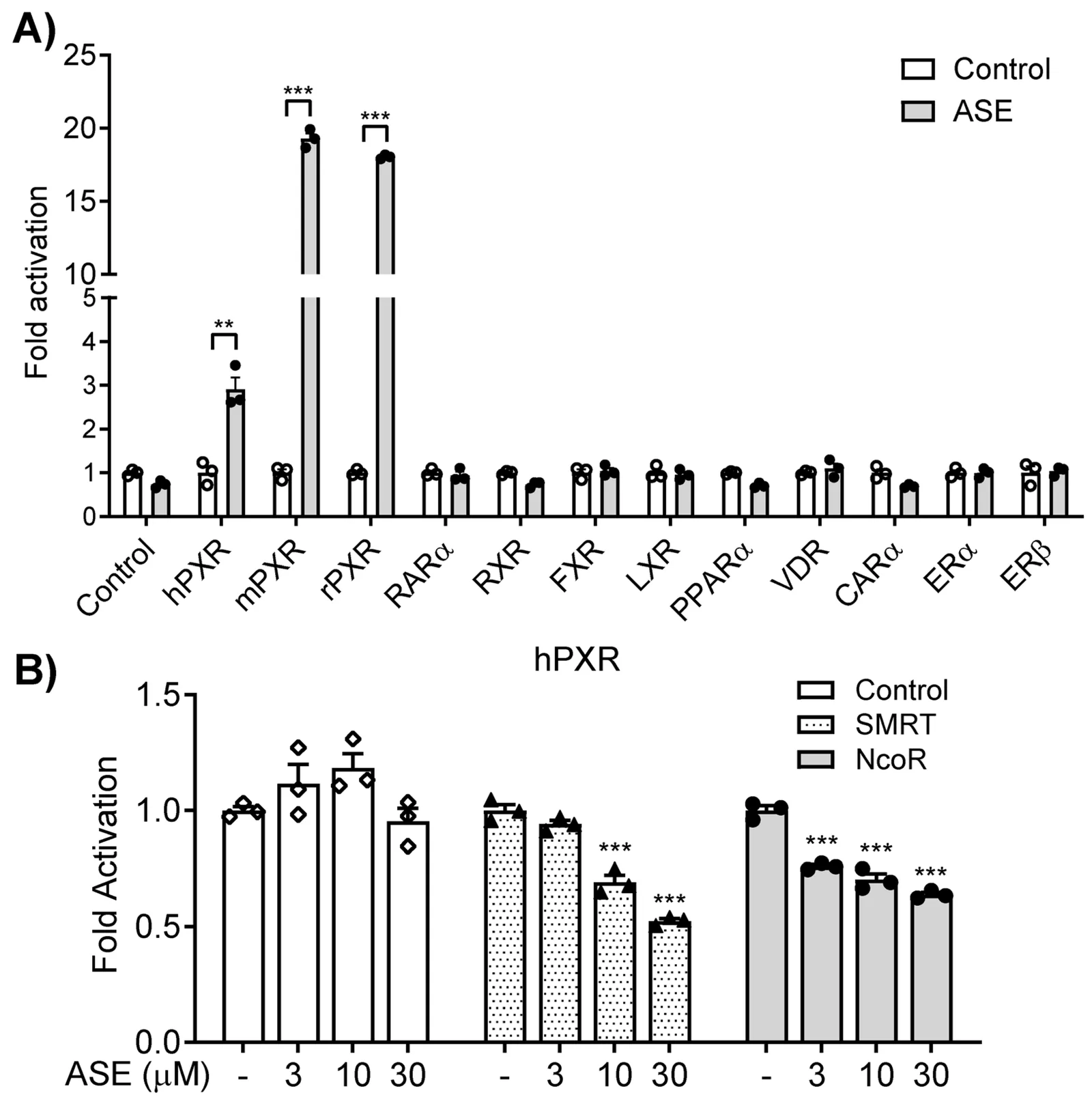
Examine whether ASE is a selective PXR agonist. (**A**) HepG2 cells were transfected with a GAL4 reporter and a series of GAL4 constructs in which the GAL4 DNA-binding domain (DBD) was fused to the ligand-binding domain (LBD) of the indicated nuclear receptors, followed by treatment with 20 µM of ASE or DMSO control for 24 h (n = 3, two-sample, two-tailed Student’s *t*-test, ** *p* < 0.01 and *** *p* < 0.001 compared to control group). (**B**) HepG2 cells were transfected with a GAL4 reporter, a VP16-hPXR vector, and either a GAL4 DBD control vector or GAL4 DBD fused to the receptor interaction domains of PXR co-repressors SMRT or NCoR (GAL4-SMRT or GAL4-NCoR), followed by incubation with DMSO vehicle control or ASE at the indicated concentrations for 24 h (n = 3, one-way ANOVA, Dunnett’s *t* test for multiple comparisons to control, *** *p* < 0.001). Reporter activity was normalized to the β-gal transfection controls and expressed as Relative Light Units (RLUs) per OD_420_ β-gal per minute to facilitate comparisons between plates. Fold activation was calculated relative to DMSO vehicle controls. Error bars represent ± SEM.

To determine whether ASE was a PXR-specific agonist, we tested the ability of ASE to activate other nuclear receptors, including rat PXR (rPXR), human retinoid acid receptor (RAR)α, retinoid X receptor (RXR), farnesoid X receptor (FXR), liver X receptor (LXR), peroxisome proliferator-activated receptor (PPAR)α, vitamin D receptor (VDR), constitutive androstane receptor (CAR)α, estrogen receptor (ER)α, and ERβ. ASE activated PXRs but did not activate any of the other nuclear receptors tested, indicating that ASE may act as a selective PXR agonist. Notably, ASE exhibited higher activities toward rodent PXRs than human PXR (Figure 2A). To further investigate the agonistic effects of ASE on PXR, we examined whether ASE modulates the interaction between human PXR (hPXR) and nuclear receptor co-repressors using a mammalian two-hybrid assay, given the critical role of nuclear receptor co-regulators in regulating receptor activation. Our results showed that, in the absence of ASE, unliganded hPXR interacted with the co-repressors silencing mediator of retinoid and thyroid hormone (SMRT) and nuclear receptor co-repressor (NCoR) (Figure 2B), whereas, in the presence of ASE, hPXR dissociated from SMRT or NCoR in a dose-dependent way (Figure 2B), suggesting the induced hPXR transcriptional activation by ASE.

### 2.2. Key Amino Acid Residues Within hPXR Required for ASE’s Agonistic Activity

**Figure 3:**
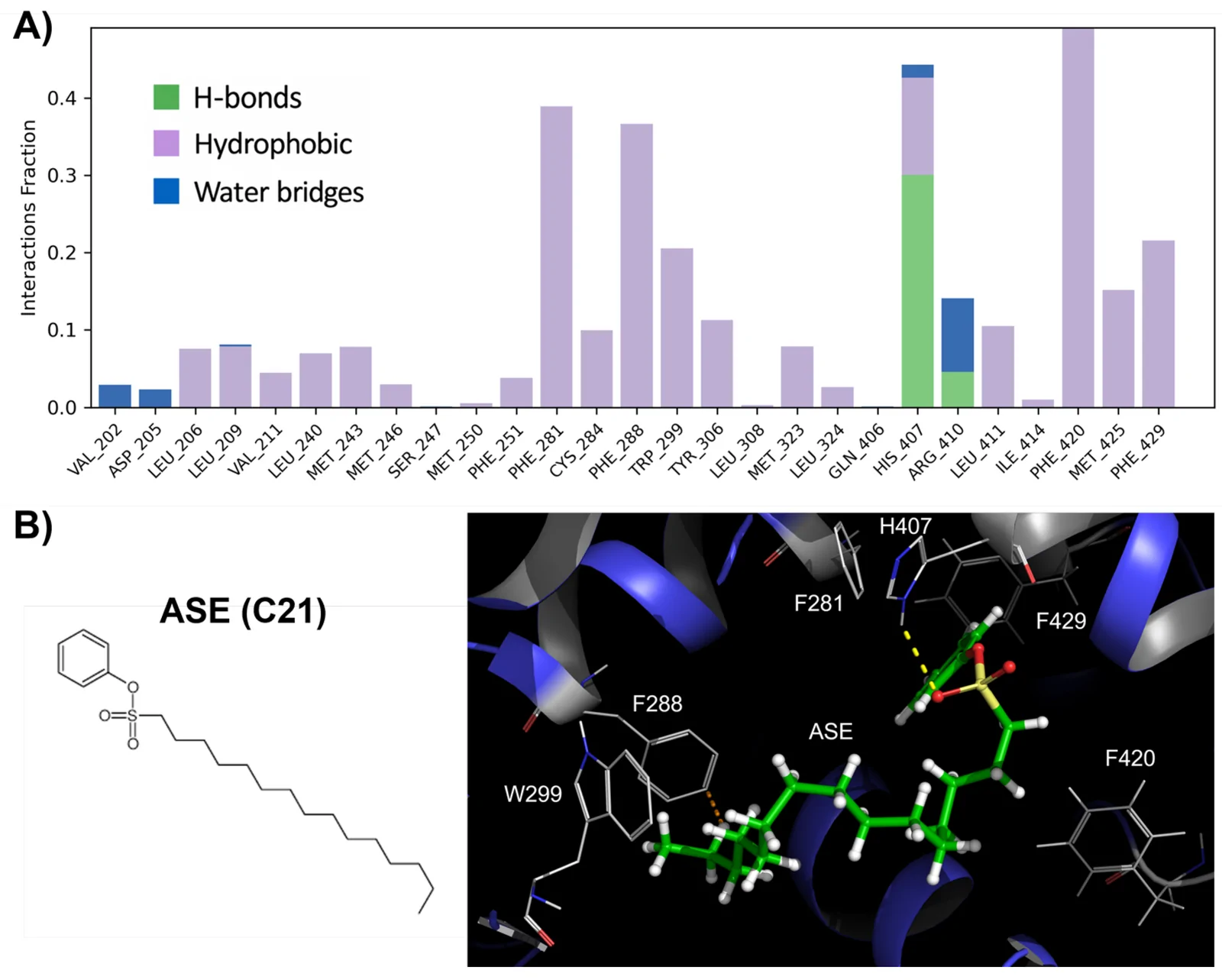
The hPXR/ASE interactions determined through docking and subsequent MD simulation. (**A**) The interaction fraction of amino acid residues from hPXR in contact with ASE (C21) are shown through the course of the MD simulation (200 ns). The type of interaction, such as H-bond, hydrophobic, ionic, water bridge, and halogen bonds, are given in the legend. (**B**) The best docked pose using Schrodinger’s GLIDE software is shown between hPXR (PDB: 3HVL) and ASE (C21). Selected residues from a subsequent MD simulation that were shown to interact with hPXR residues are shown in grey while ASE is in green. H-bonds are shown as a yellow dashed line, and bad interactions are shown as an orange dashed line. The figure was made with PyMOL v3.1.3 [27].

**Figure 4:**
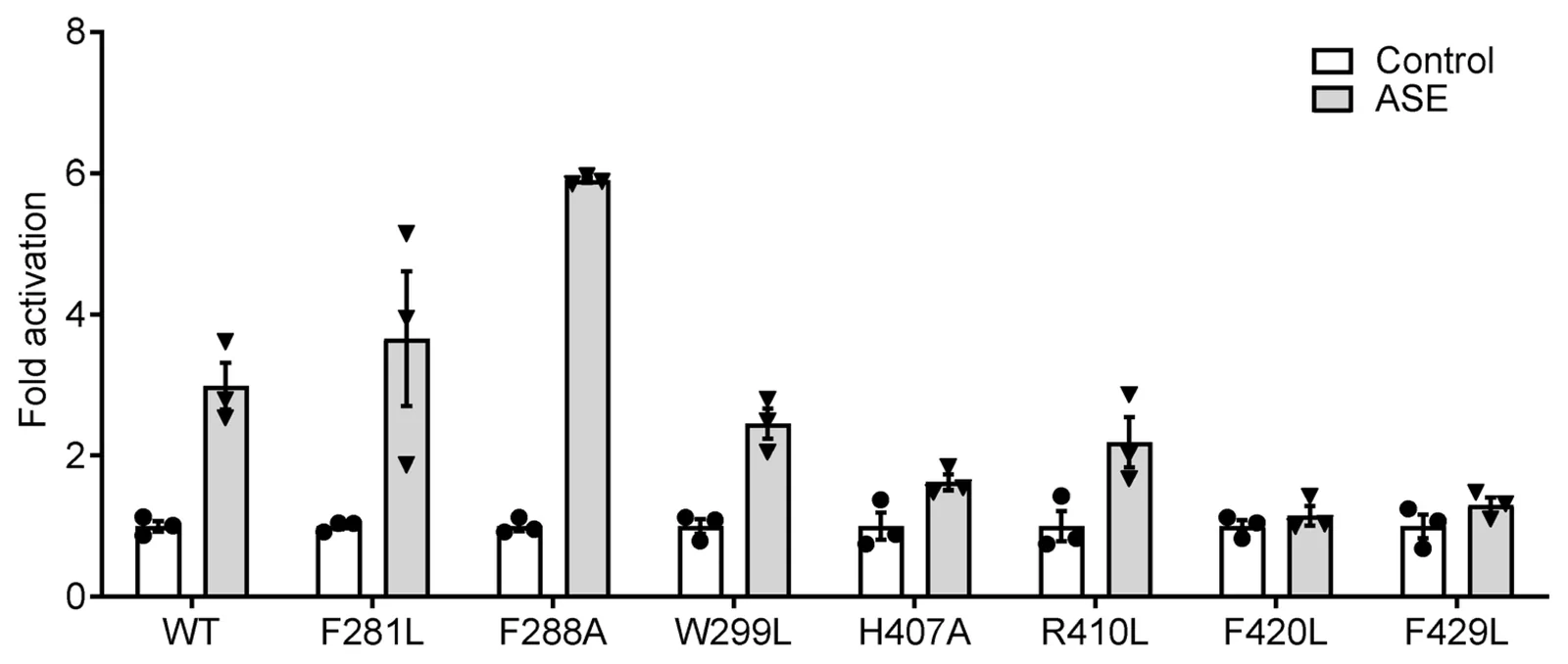
Key residues of PXR LBD required for the agonistic activity of ASE. HepG2 cells were co-transfected with a full-length WT hPXR plasmid or the indicated hPXR mutant plasmids, together with CYP3A4-luc reporter and β-gal plasmid, followed by treatment with control medium or medium containing 20 μM of ASE for 24 h. Reporter activity was normalized to the β-gal transfection controls and expressed as Relative Light Units (RLUs) per OD_420_ β-gal per minute to facilitate comparisons between plates. Fold activation was calculated relative to DMSO vehicle controls. Error bars represent ± SEM.

To provide insight into the molecular interactions between hPXR and ASE, we docked the representative C21 congener of ASE (ASE (C21)) to an available crystal structure of hPXR (PDB: 3HVL). The best docking pose is provided in Figure 3B and shows a H-bonding interaction between the sulfonyl oxygen of ASE (C21) and the protonated nitrogen His407 of hPXR. Additionally, the docking predicted a bad interaction between the ASE (C21) hydrocarbon tail and Phe288 (Figure 3B; orange dashed line). A GLIDE docking score of −7.1 kcal/mol for ASE (C21) was determined and suggests a strong binding affinity. Using the docked pose as a starting point, the hPXR/ASE complex was then subjected to a molecular dynamics (MD) simulation using the Desmond program within the Schrödinger software suite. The hPXR/ASE simulation interactions were then assessed and shown in Figure 3A. These results predicted several hPXR/ASE interactions where His407 and Phe420 were among the highest predicted interactions. Next, we verified the docking-predicted amino acids within hPXR ligand-binding domain (LBD) required for ASE’s agonistic activity using the site-directed mutagenesis and cell-based transfection assays. Our results showed that ASE’s agonistic activity was abolished by Phe420Leu and Phe429Leu mutations and was weakened by Trp299Leu, His407Ala, and Arg410Leu mutations (Figure 4). The Phe288Ala mutant increased hPXR’s activity by ASE eliminating the bad interaction between ASE and Phe288. Taken together, coupling the computational strategy with site-directed mutagenesis we established the key amino acids within PXR’s binding pocket that are essential for the agonistic effects of ASE.

### 2.3. Impacts of ASE on Plasma Lipid Levels in Wildtype Mice

To determine the optimal ASE dose for inducing PXR activity in mice while minimizing toxicity, male C57BL/6 wildtype (WT) mice were initially administered ASE orally by gavage at doses of 3 or 10 mg/kg body weight (BW) per day for seven consecutive days. QPCR analysis showed that the established mouse PXR target gene *CYP3A11* was significantly induced in liver by ASE at 10 mg/kg BW, whereas no significant induction was observed at 3 mg/kg BW (Supplementary Figure S2A). ASE exposure at either dose did not alter the weights of major organs, including liver, spleen, and kidney (Supplementary Figure S2B–D), which suggested that the dose of 10 mg/kg BW was optimal to activate PXR signaling with no toxicity to major organs. We next employed the established specific PXR antagonist RES to investigate if the potential ASE roles were mediated by PXR [28]. To determine the optimal dose of the PXR antagonist RES, mice were administered RES at two different doses (45 or 75 mg/kg/day) by oral gavage daily in combination with ASE at 10 mg/kg BW. QPCR analysis showed that hepatic expression of the PXR target gene *CYP3A11* was significantly reduced by RES at both 45 and 75 mg/kg BW per day. (Supplementary Figure S3).

**Figure 5:**
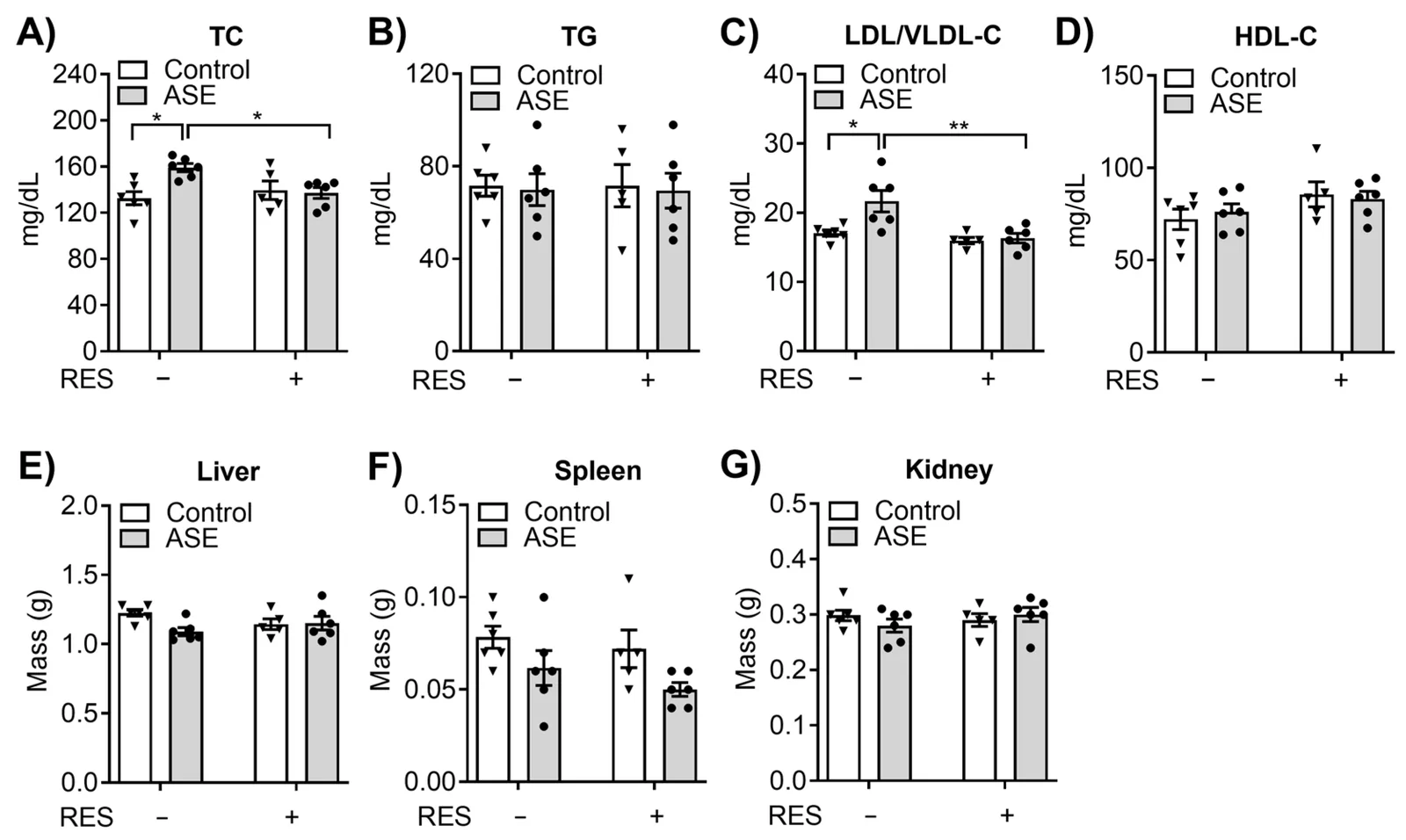
Effects of ASE exposure on plasma lipid levels in wildtype mice. Eight-week-old male WT mice were treated with vehicle control, ASE (10 mg/kg/day), and/or PXR-specific antagonist RES (45 mg/kg/day) by oral gavage for 1 week on AIN76 diet. Fasting plasma total cholesterol (**A**) and triglyceride (**B**) levels were measured using enzymatically colorimetric assays (n = 5–6, two-way ANOVA, Bonferroni multiple comparisons test for multiple comparisons, * *p* < 0.05). (**C**,**D**) Lipoprotein fractions (LDL/VLDL and HDL) were isolated, and the cholesterol levels of each fraction were measured (n = 5–6, two-way ANOVA, Bonferroni multiple comparisons test for multiple comparisons, * *p* < 0.05 and ** *p* < 0.01). Major organs, including liver (**E**), spleen (**F**), and kidney (**G**), were weighed under anesthesia. Data are presented as mean ± SEM.

To investigate whether ASE exposure could change plasma lipid levels in vivo, we treated C57BL/6 WT male mice with ASE (10 mg/kg/day) in combination with the PXR antagonist RES (45 mg/kg/day) for one week on AIN76 diet, which has been previously used in studies of dyslipidemia and atherosclerosis in mice [9,11]. Our results suggested that short-term ASE exposure significantly increased plasma total cholesterol levels, which were revoked by PXR antagonist RES, implying that PXR contributed to this increase (Figure 5A). Consistent with this finding, ASE-treated WT mice exhibited significantly higher levels of atherogenic VLDL and LDL cholesterol compared with control mice, whereas these increases were not observed in mice co-treated with RES, further supporting a role for PXR signaling in ASE-mediated regulation of plasma cholesterol levels (Figure 5C). In contrast, ASE treatment did not significantly affect plasma HDL cholesterol levels (Figure 5D), plasma triglyceride levels (Figure 5B), or the weights of major organs (Figure 5E–G). Collectively, these findings suggested that short-term ASE exposure increases atherogenic plasma cholesterol levels consistent with a PXR-mediated effect.

### 2.4. Effects of ASE Exposure on Intestinal Lipogenic Gene Expression in WT Mice

**Figure 6:**
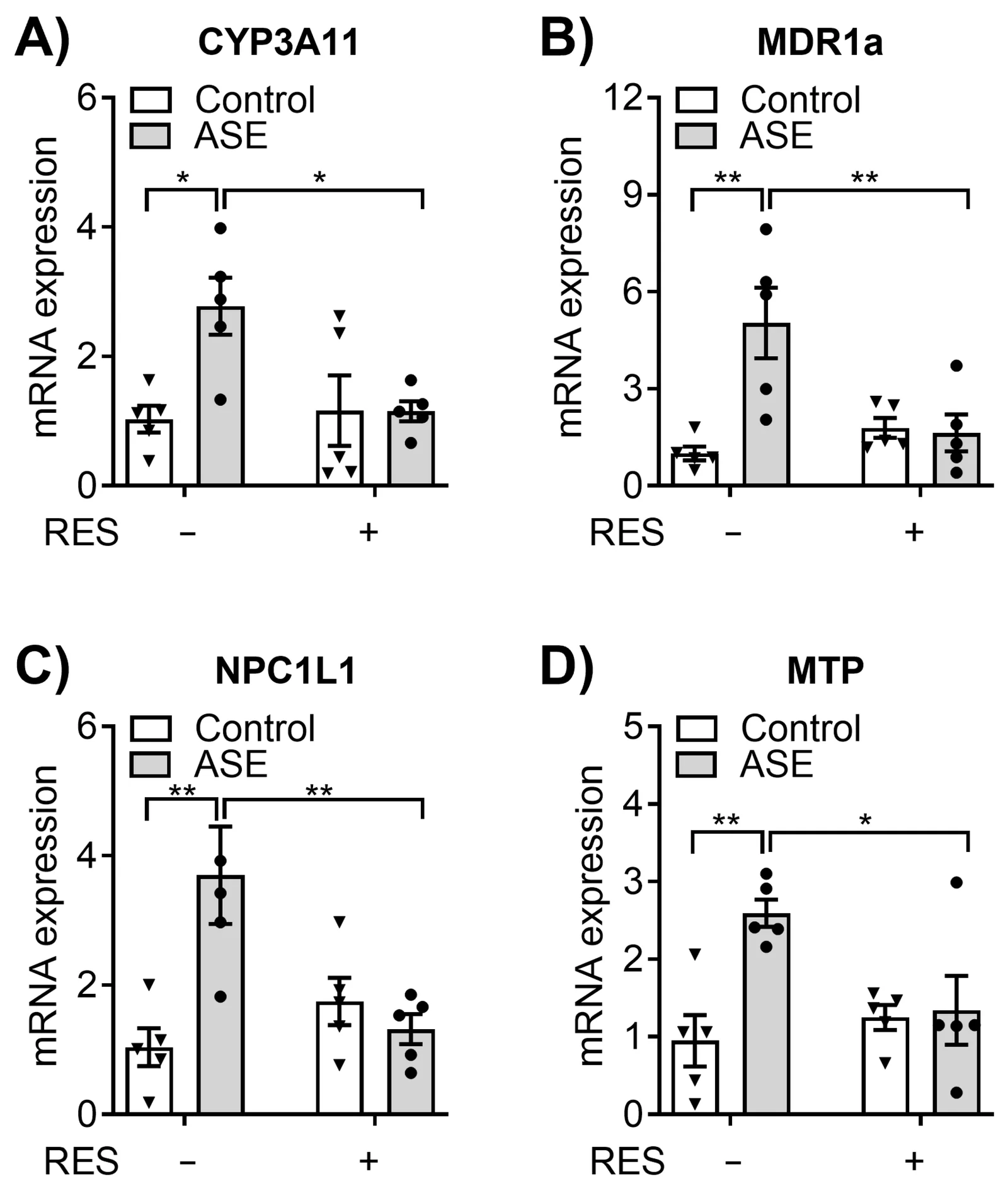
The influences of ASE exposure on intestinal lipogenic gene expression in WT mice. Eight-week-old male WT mice were administered ASE (10 mg/kg/day) and/or RES (45 mg/kg/day) by oral gavage for 7 days on AIN-76 diet. (**A**,**B**) The intestinal expression of PXR target genes was measured by QPCR (n = 5, two-way ANOVA, Bonferroni multiple comparisons test for multiple comparisons, * *p* < 0.05 and ** *p* < 0.01). (**C**,**D**) The expression of key intestinal cholesterol transporters was analyzed by QPCR (n = 5, two-way ANOVA, Bonferroni multiple comparisons test for multiple comparisons, * *p* < 0.05 and ** *p* < 0.01). Data are presented as mean ± SEM.

To determine whether ASE increased circulating cholesterol levels through PXR signaling, we analyzed the expression of established PXR target genes by QPCR in C57BL/6 WT mice orally administered ASE (10 mg/kg/day) with or without RES (45 mg/kg/day) for 7 days. ASE treatment significantly induced the mRNA expression of two PXR target genes, *CYP3A11* and *MDR1a*, in both the intestine (Figure 6A,B) and liver (Supplementary Figure S4A,B) in the absence of RES. However, this induction was not observed in RES-treated mice, suggesting the activation of PXR signaling pathway by ASE in vivo. We next examined whether ASE altered the expression of intestinal lipogenic genes involved in lipid homeostasis that are also direct transcriptional targets of PXR. ASE treatment increased the mRNA expression of the key intestinal cholesterol transporters Niemann–Pick C1-Like 1 (NPC1L1) [8] and microsomal triglyceride transfer protein (MTP) [29] in WT mice, whereas these effects were abolished in PXR-inhibited mice (Figure 6C,D). Taken together, these findings suggested that ASE exposure increased plasma atherogenic cholesterol levels, at least in part, by inducing the expression of key intestinal PXR-regulated lipogenic genes.

### 2.5. Evaluate the Potential Roles of ASE in Cholesterol Uptake by Human Intestinal Cells

**Figure 7:**
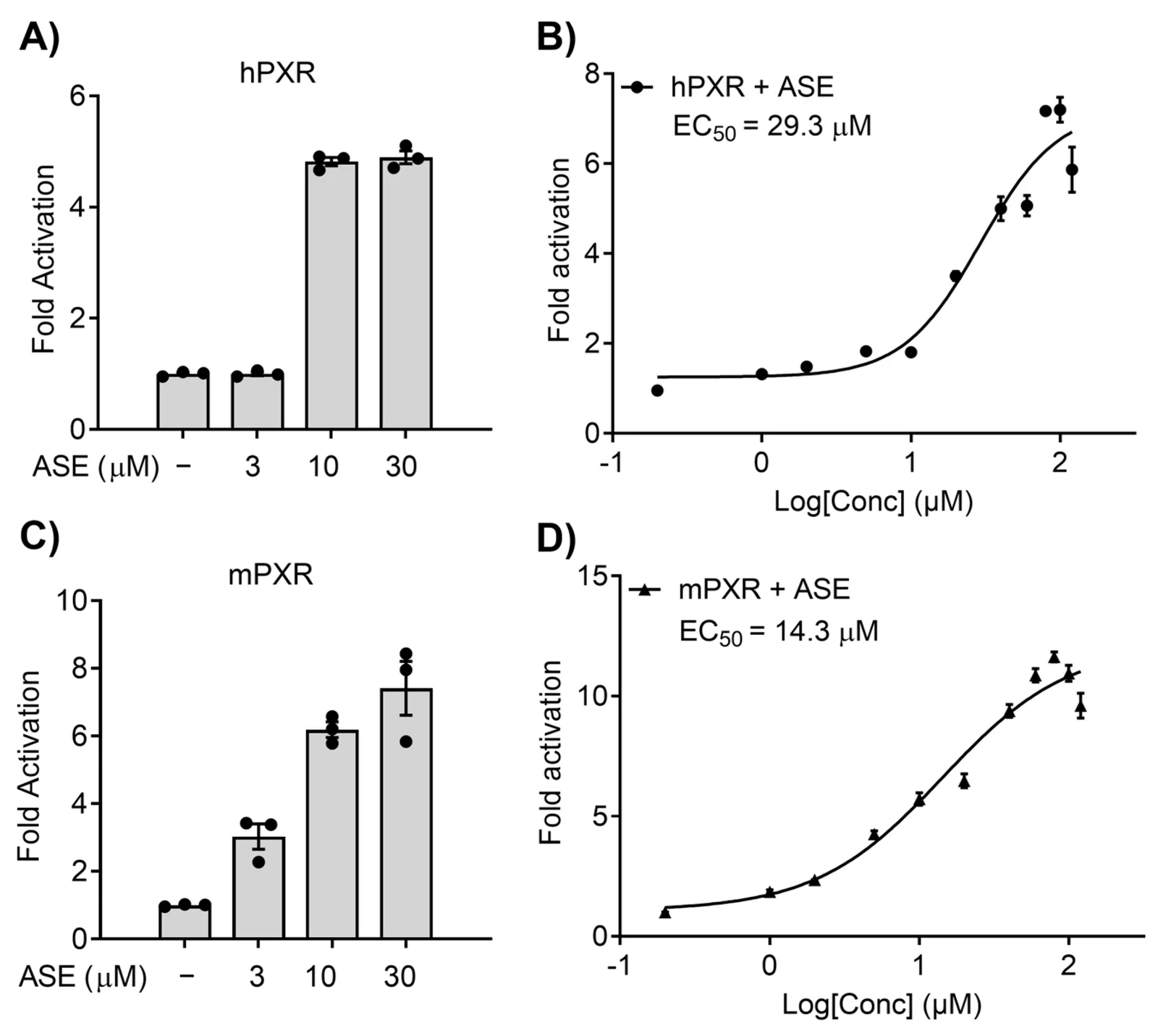
The effects of ASE on PXR activity in human intestinal LS180 cells assessed by transfection assay. Human LS180 intestinal cells were transfected with β-gal control plasmid together with (**A**,**B**) full-length hPXR expression plasmid and hPXR-responsive CYP3A4-luc reporter plasmid, or (**C**,**D**) full-length mPXR plasmid and mPXR-responsive (CYP3A2)_3_-luc reporter plasmid. Cells were subsequently treated with ASE (**A**,**C**) at the indicated concentrations or (**B**,**D**) at concentrations ranging from 0.2 to 120 µM for 24 h (n = 3). Reporter activity was normalized to the β-gal transfection control and expressed as Relative Light Units (RLUs) per OD_420_ β-gal per minute to facilitate comparisons between plates. Fold activation was calculated relative to vehicle (DMSO) controls. Data are presented as mean ± SEM.

**Figure 8:**
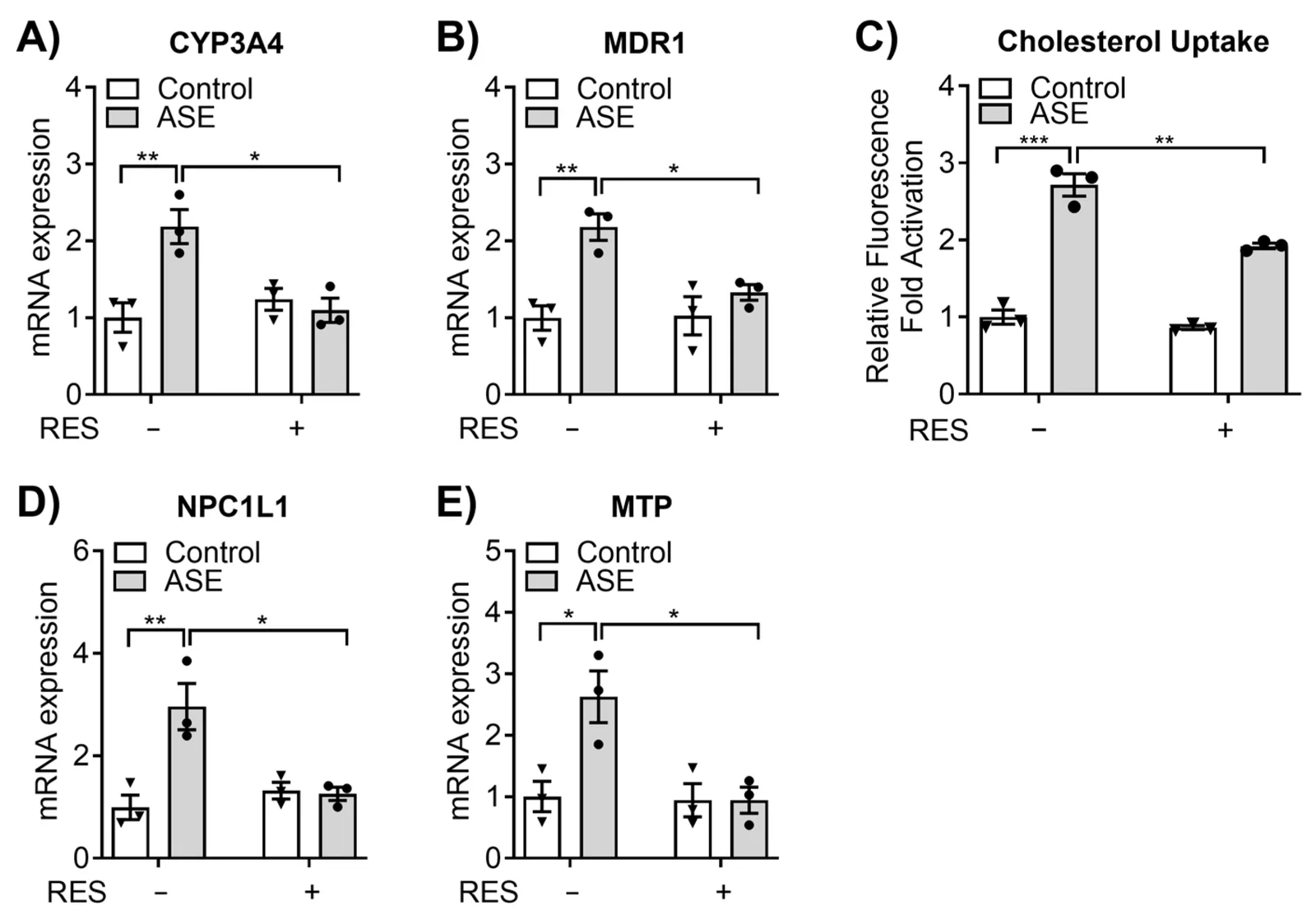
The impacts of ASE exposure on cholesterol uptake in human intestinal LS180 cells. Human LS180 cells were treated with vehicle control or ASE (20 µM) in the presence or absence of PXR antagonist RES (50 µM) in serum-free culture media for 24 h. (**A**,**B**) The expression of PXR target genes was measured by QPCR (n = 3, two-way ANOVA, Bonferroni multiple comparisons test for multiple comparisons, * *p* < 0.05 and ** *p* < 0.01). (**C**) Cells were co-incubated with GFP-labeled cholesterol for 24 h, and cholesterol uptake was quantified by measuring fluorescence at Ex/Em = 485/535 nm. Results are presented as fold changes in relative fluorescence units compared with the DMSO vehicle control (n = 3, two-way ANOVA, Bonferroni multiple comparisons test for multiple comparisons, ** *p* < 0.01 and *** *p* < 0.001). (**D**,**E**) The expression of key intestinal cholesterol transporters was analyzed by QPCR (n = 3, two-way ANOVA, Bonferroni multiple comparisons test for multiple comparisons, * *p* < 0.05 and ** *p* < 0.01). Data are presented as mean ± SEM.

To investigate the potential mechanisms underlying the ASE-induced increase in plasma cholesterol levels, we used human intestinal LS180 cells to examine whether ASE affects cholesterol uptake through intestinal PXR signaling. We first performed a cell-based transfection assay in LS180 cells to determine the ability of ASE to activate human PXR (hPXR) and mouse PXR (mPXR). ASE treatment increased the reporter activity of both hPXR (Figure 7A) and mPXR (Figure 7C) in a concentration-dependent manner. Dose response curve analysis revealed that the EC_50_ for ASE-mediated activation of hPXR and mPXR were 29.3 μM and 14.3 μM, respectively (Figure 7B,D). Interestingly, ASE induced higher PXR activities in human intestinal LS180 cells (Figure 7) than human hepatic HepG2 cells (Figure 1). A previous study demonstrated that treatment with 50 µM RES effectively attenuated rifampicin-induced PXR activity in hPXR-overexpressing human intestinal LS174T cells without affecting cell viability [28]. Therefore, 50 µM of RES was used to inhibit ASE-induced PXR activities in human intestinal LS180 cells in the presence of 20 µM of ASE for one day. QPCR analysis showed that ASE treatment significantly increased the expression of the human PXR target genes *CYP3A4* and *MDR1*, whereas this induction was attenuated by RES co-treatment (Figure 8A,B), which indicated that ASE-mediated PXR activation in human intestinal LS180 cells could be inhibited by RES. To determine whether ASE altered cholesterol uptake in human intestinal cells, LS180 cells were incubated with GFP-labeled cholesterol for 24 h. Notably, cholesterol uptake was significantly increased in ASE-treated cells, whereas this effect was not observed in cells co-treated with both ASE and RES, suggesting that ASE enhanced cholesterol uptake through PXR signaling (Figure 8C). Consistently, QPCR analysis demonstrated that ASE treatment increased the expression of the intestinal cholesterol transporters *NPC1L1* and *MTP* in LS180 cells, whereas this induction was abolished by RES co-treatment (Figure 8D,E). In sum, our data suggested that ASE exposure increased cholesterol uptake by human intestinal cells, potentially through PXR-mediated induction of key intestinal cholesterol transporters.

## 3. Discussion

Although associations between environmental chemicals and CVD have begun to emerge [2,3,30,31,32,33,34], the underlying mechanisms are still elusive. As a substitute for phthalates, ASE has been widely used in PVC product such as medical tubing [14]; however, it remains unclear whether ASE exerts adverse cardiovascular effects and which nuclear receptor(s) may be activated by ASE to mediate its cellular signaling. In the present study, we identify ASE as a PXR agonist for the first time and explore the key amino acid residues within human PXR ligand-binding domain (LBD) interacting with ASE. Our findings further demonstrated that short-term ASE exposure increased plasma atherogenic cholesterol levels, which may be partly attributable to enhanced intestinal cholesterol uptake mediated by PXR.

The transcriptional regulators, including nuclear receptors (NRs), control basal machinery assembly and regulate gene expression. The ligand binding pocket of PXR is large enough to bind to diverse ligands to regulate PXR’s transcriptional activity [35]. So far, it is unknown whether ASE could activate PXR in humans or animals. In our study, ASE was found to induce PXR activities of human and mouse PXR reporters using transfection assays in both human hepatic and intestinal cells (Figure 1 and Figure 7). Moreover, ASE only activated PXRs rather than the other NRs we tested, suggesting ASE as a PXR selective agonist (Figure 2A). Some PXR agonists possess tissue-specific properties on PXR activation. As a potent PXR agonist, tributyl citrate (TBC) can only activate intestinal PXR but not hepatic PXR [8]. In contrast, Tocotrienols can upregulate expression of PXR target gene *CYP3A4* in human primary hepatocytes but not in intestinal cells [36]. Intriguingly, ASE displayed more intense ability to activate PXR in intestinal cells than hepatic cells based on cell-based transfection assay, which was plausibly caused by the co-regulator-specific effects or differential catalyzation in cells [8]. The AF-2 region of the LBD undergoes a conformational change upon ligand binding, resulting in the dissociation of corepressors and subsequent activation of target gene transcription [37]. Thus, we used the mammalian two-hybrid assay to examine how ASE affected the interaction between hPXR and corepressors, which is critical for nuclear receptor activity. Our data suggested that ASE promoted the dissociation of PXR from its co-repressors NCoR and SMRT in a dose-dependent way (Figure 2B). Furthermore, we determined a computationally predicted structure between ASE and hPXR using docking, which was then tested experimentally using site-directed mutagenesis. The highly conserved residue Trp299 lined a hydrophobic region within the PXR ligand binding pocket where the residues His407, Arg410, Phe420, and Phe429 interacted with ASE through hydrogen bonding and non-polar interactions [38]. Intriguingly, the mutation of Phe288Ala increased hPXR’s activity by ASE, by potentially increasing the size of the hPXR ligand pocket to better accommodate the rather large ASE structure. A clash between the hydrocarbon tail of ASE and Phe288 is predicted in the docking, shown as a bad interaction (the orange dashed line in Figure 3). To our knowledge, this study is the first to establish a link between ASE exposure and the pro-atherogenic PXR signaling pathway and to identify the key amino acid residues critical for the interaction between ASE and PXR.

Human biomonitoring data establishing ASE concentrations in serum/plasma or tissue appear to be very limited; therefore, it would be difficult to justify a particular cellular concentration that is directly equivalent to human internal exposure. Concentrations were selected to encompass low-dose exposure-oriented conditions and higher concentrations suitable for mechanistic characterization. Based on our MTT assay and cell transfection assay results, 20 µM of ASE could significantly activate PXR without altering cell growth, so we used ASE at a dose of 20 µM for our cholesterol uptake assay. ASE concentrations were selected to span low micromolar concentrations for in vitro mechanistic characterization, while the in vivo dose range was selected to encompass low exposure-oriented doses and doses below concentrations associated with overt systemic toxicity in repeated-dose animal studies. The available human exposure estimates are on non-dietary dust ingestion (0.08–0.86 µg/kg/day), whereas the existing repeated-dose animal toxicity studies used doses tens to hundreds of mg/kg/day [16]. Our QPCR data suggested that ASE at 10 mg/kg BW, but not at 3 mg/kg BW, could induce PXR signaling in mice without affecting the major organ weights. Thus, ASE at 10 mg/kg/day was selected for our in vivo studies. It is distinguishable that the strongest available human evidence is currently based on external exposure through dust, not measured circulating ASE concentrations. Hence, valid risk assessment remains difficult because toxicological data and exposure through other pathways, including food, remain limited.

As a xenobiotic sensor, PXR mainly functions in the liver and intestine by activating the genes required for xenobiotic metabolism, such as cytochrome P450s (CYP), conjugating enzymes (e.g., glutathione transferase (GST)), and ABC family transporters (e.g., multidrug resistance 1 (MDR1)) [39,40]. In this study, the PXR target genes *CYP3A11* and *MDR1* were upregulated in both the liver and intestine of mice fed ASE, suggesting the effective activation of PXR signaling. In addition, PXR has been associated with lipid homeostasis [10] and also acts as a sensor of endogenous lipophilic metabolites, such as the toxic bile acid lithocholic acid [41] and other cholestatic bile acids [42], regulating genes involved in bile acid synthesis, transport and metabolism. In this dual xenobiotic/endobiotic-sensing function, PXR has been suggested to play a more comprehensive role in cardiometabolic diseases [43], thereby providing further mechanistic support for the crosstalk between structurally unrelated exogenous and endogenous ligands. For example, chronic activation of PXR by a potent mouse PXR ligand pregnenolone 16α-carbonitrile increased plasma total and LDL cholesterol levels in WT mice, but not in PXR-deficient mice [12]. The oral toxicokinetic of ASE were evaluated in Wistar rats, revealing a half-life of approximately 8 days in adipose tissue, with 20–30% of the administered dose excreted in feces within 24 h [21]. It is possible that ASE has multidirectional impacts on the cardiovascular system. In our animal models, ASE treatment increased the plasma total cholesterol level and atherogenic LDL/VLDL cholesterol level, which was abolished by PXR antagonist RES treatment (Figure 5). HDL synthesis occurs in the liver and the intestines and is taken up by liver mainly through cholesterol ester transfer protein pathway [44,45], leading to the transfer of cholesterol in HDL to the liver and the elimination of HDL from the circulation [46]. In most mammalian cells, the glycerol-3-phosphate pathway is the principal route for the synthesis of triglyceride [47]. In the ASE fed mice, the levels of HDL cholesterol and triglycerides were not changed; therefore, it is possible that ASE-induced PXR signaling did not affect/interact with the biochemical pathways associated with the synthesis of HDL and/or triglyceride. These data suggest that ASE potentially induced hypercholesterolemia in mice by PXR in a direct or indirect way.

Altered intestinal cholesterol absorption has been associated with plasma lipid levels and cardiovascular disease in both rodents and humans. Intestinal lipid transportation plays an important role in maintaining lipid homeostasis. In human intestinal LS180 cells, our data suggests that ASE incubation increased the cholesterol uptake, which was inhibited by the natural PXR antagonist RES (Figure 8C). PXR has been shown to regulate multiple intestinal and hepatic genes involved in lipid homeostasis across various animal models [8,10,23,24,40]. Although the LBD of PXR exhibits species-specific characteristics, the DNA-binding domain (DBD) of PXR is highly conserved between humans and rodents [10]. ASE-mediated PXR activation significantly increased the expression of two key lipogenic genes, *NPC1L1* and *MTP*, in both WT mice and human intestinal LS180 cells. Both genes are direct transcriptional targets of PXR and play important roles in intestinal cholesterol homeostasis. Notably, NPC1L1 is a key intestinal cholesterol transporter, and NPC1L1-deficient mice exhibit markedly reduced intestinal cholesterol absorption and plasma LDL cholesterol levels, as well as resistance to diet-induced hypercholesterolemia and atherosclerosis. Therefore, NPC1L1 has been established as a therapeutic target of ezetimibe, a cholesterol-lowering drug that inhibits cholesterol absorption [48,49]. Intestinal MTP is important for lipid absorption and lipoprotein assembly [50]. Overexpression of MTP induced hyperlipidemia and atherosclerosis in mice [51,52], whereas inhibition or deletion of MTP has been shown to reduce plasma lipid levels and attenuate the development of atherosclerosis in mice [53,54]. Findings from the present study will provide a foundation for future investigation into the potential effects and underlying mechanisms of long-term ASE exposure on cardiometabolic disease.

Hypercholesterolemia is a major risk factor for atherosclerosis [55]. It has been established that estrogen has athero-protective effects in animals and humans [56,57,58]. To exclude the athero-protective roles of estrogen, we used male mice to investigate how ASE affected dyslipidemia. Our results suggested that ASE did not activate estrogen receptors; however, it is crucial to examine whether ASE could impact the lipid homeostasis in female mice or estrogen receptor-deficient mice in future. Chronic activation of PXR may enhance lipid uptake by macrophages, thereby promoting foam cell formation and contributing to atherogenesis in hyperlipidemic ApoE^−^ mice [11,12]. In this study, we only investigated the effects of short-term exposure to ASE on plasma lipid levels in mice; however, further studies are necessary to explore how long-term ASE exposure could affect atherosclerotic development. For example, we will test whether chronic PXR activation by ASE exposure increases atherosclerosis in apolipoprotein E-deficient (ApoE^−^) mice. PXR^−^ApoE^−^ mice and ApoE^−^ mice will be fed an atherosclerosis diet supplemented with ASE for 12 weeks to determine the contribution of PXR towards the potential atherogenic effects of ASE in vivo. Atherosclerosis will be evaluated using cross-sectional aortic root, brachiocephalic artery, and whole aorta analysis, and the internal ASE concentrations will be correlated to atherosclerotic lesion sizes. Transcriptomic profiles in liver, intestine, and macrophage will be compared between male and female mice using RNA sequencing. We will also determine the effects of ASE-mediated PXR activation on macrophage foam cell formation and other macrophage functions involved in atherosclerosis. Furthermore, PXR activation by certain ligands also exhibits tissue-specific patterns. For instance, Tocotrienol, a member of vitamin E family, can activate PXR in hepatocytes but not in enterocytes [36], whereas an FDA-approved plasticizer TBC can only activate intestinal PXR but not hepatic PXR [8]. Therefore, tissue-specific PXR knockout mouse models will be necessary to further investigate the potential tissue-specific effects of ASE in the intestine, liver, and macrophages. In summary, our study provides insights into the potential molecular mechanisms by which ASE exposure may increase the risk of dyslipidemia through human PXR-mediated signaling. Our data elucidates evidence to inform future assessment of ASE on cardiovascular disease risks and provides novel mechanistic links explaining how exposure to certain environmental chemicals causes dyslipidemic effects.

## 4. Materials and Methods

### 4.1. Reagents and Plasmids

ASE (11–101–5890, Thermo Fisher Scientific, Waltham, MA, USA) and RES (R0071, TCI Chemicals, Portland, OR, USA) were dissolved in dimethyl sulfoxide (DMSO). The plasmids used in this study were previously described [59] and included human (h) and mouse (m) PXR expression vectors; PXR-dependent CYP3A4 promoter reporter (CYP3A4XREM-Luciferase) and CYP3A2 promoter reporter ((CYP3A2)_3_-luciferase) constructs; β-galactosidase (β-gal) expression vector; GAL4 DNA-binding domain (DBD)-linked nuclear receptor ligand-binding domain (LBD) vectors; VP16-hPXR, GAL4 DBD-linked nuclear receptor co-repressors constructs, including SMRT (Silencing mediator of retinoid and thyroid hormone receptors) and NCoR (Nuclear receptor co-repressor); and GAL4-responsive reporter construct (MH100-Luciferase).

### 4.2. Cell Culture and Transfection Assay

The human hepatic cell line HepG2 (ATCC, HB-8065) and human intestine epithelial cell line LS180 (CL-187, ATCC, Manassas, VA, USA) was obtained from the American Type Culture Collection. Both cell lines were cultured in DMEM supplemented with 10% fetal bovine serum (FBS) at 37 °C in a humidified atmosphere containing 5% CO_2_. MTT assay (4890–050-K, R&D Systems, Minneapolis, MN, USA) was used to examine if ASE at the doses of 3, 10, 30, or 100 µM affect the cell proliferation of HepG2 and LS180 cells (Supplementary Figure S1). Transfection assays were performed as previously described [9]. Briefly, cells at approximately 80% confluency in 24-well plates were transiently transfected with the indicated expression plasmids and the corresponding luciferase reporter plasmids, together with β-gal control plasmids, using FuGENE 6 (E2691, Promega Corporation, Madison, WI, USA) in serum-free DMEM. For mammalian two-hybrid assays, HepG2 cells were transfected with MH100-Luciferase GAL4 reporter, VP16-hPXR, and co-repressor plasmids [60,61] for 24 h, followed by treatment with the indicated chemicals or DMSO vehicle for an additional 24 h in serum-free DMEM. Cell lysates were subsequently collected for luciferase and β-galactosidase assays. For β-galactosidase assays, cell lysates were incubated with β-gal substrate solution at 37 °C for 30 min, and then the reaction was terminated by addition of 1M Na_2_CO_3_. Absorbance was measured at 420 nm (OD_420_) using a Synergy H1 Hybrid Reader (11120533, Agilent BioTek Instruments, Winooski, VT, USA). Luciferase assays were performed as manufacturer’s instructions (Promega Corporation, E1531) using the same reader. Reporter activity was normalized to the β-gal transfection control and expressed as normalized Relative Light Units (RLUs) per OD_420_ β-gal per minute to facilitate comparisons between plates. Fold activation was calculated relative to the corresponding solvent control. EC_50_ values were calculated by nonlinear curve fitting using GraphPad Prism software v11.0.2.

### 4.3. Molecular Docking and MD Simulation

The hPXR crystal structure with ligand ([2-(3,5-di-tert-butyl-4-hydroxy-phenyl)-1-(diethoxy-phosphoryl)-vinyl]-phosphonic acid diethyl ester) bound was obtained from the Protein Data Bank (PDB: 3HVL) [62]. All computations and preparations were conducted using the Schrödinger software suite (New York, NY, USA). hPXR was prepared for computational work using the Protein Preparation Wizard followed by energy minimization using the OPLS4 force field. The docking grid was prepared with the Grid Generation program using the location of the original ligand. The representative C21 congener of ASE (ASE (C21)) was prepared using LigPrep and docked using the GLIDE SP docking algorithm. After docking, the hPXR/ASE complex, in its original dimeric form, was prepared for a molecular dynamics (MD) simulation using the System Setup program using the OPLS4 force field with the SPC explicit water model in an orthorhombic simulation box (about 83,000 atoms) and neutralized by the addition of Na^+^ ions. Following the standard Desmond relaxation protocol, molecular dynamics simulations were performed for 200 ns in the NPT ensemble at 300 K and 1.01 bar. No partial charges for ASE were derived outside of this program. Subsequently, the hPXR/ASE interactions were assessed using the Simulation Interactions program within the Schrödinger software suite.

### 4.4. Site-Directed Mutagenesis

The full-length human PXR expression plasmid was used as the wildtype template to generate a series of mutant constructs using the QuikChange II Site-Directed Mutagenesis Kit according to the manufacturer-supplied protocol (200524, Agilent, Santa Clara, CA, USA ). The primers used for site-directed mutagenesis are listed in Supplementary Table S1.

### 4.5. Animals

Wildtype (WT) male C57BL/6 mice were purchased from Taconic Biosciences Inc. (Germantown, NY, USA). In our previous study, mice were treated with three doses of the PXR-selective agonist tributyl citrate (TBC; 2.5, 5, or 10 mg/kg/day) for 7 days, and only the 10 mg/kg/day dose was found to activate intestinal PXR [8]. Sample size was determined based on the differences in means and standard deviation of intestinal *CYP3A11* gene expression levels between control and tributyl citrate treated WT mice [8] to estimate the minimum number of animals required to significantly differentiate two groups at a *p* value of 0.05 and a power level of 90% [63,64]. The LaMorte’s Power Calculator Microsoft Excel spreadsheet was downloaded from the website [65]. To determine an ASE dose capable of inducing PXR activation while minimizing potential toxicity, 8-week-old WT male mice were initially administered ASE at 3 or 10 mg/kg body weight/day by oral gavage for 7 days on chow diet (3 mice/group). ASE was first dissolved in DMSO and then diluted in corn oil (Fisher Science Education, S25271) to prepare the oral gavage solution. Each mouse received 100 µL of the solution daily. Blood and major tissues were subsequently collected for analysis. Our preliminary results showed that ASE at 10 mg/kg/day, but not 3 mg/kg/day, significantly activated hepatic PXR, while neither dose affected major organ weights (Supplementary Figure S2). In addition, no apparent signs of toxicity were reported in rats administered ASE by oral gavage at doses of 100 or 1000 mg/kg body weight [21]. Based on these findings, ASE at 10 mg/kg/day was selected for our subsequent studies.

To determine whether the effects of ASE were PXR-dependent, we used trans-resveratrol (RES), an established selective PXR antagonist and naturally occurring stilbenoid found in fruits [28]. Previous studies have administered RES to mice at doses ranging from 22.5 to 2500 mg/kg body weight/day [66,67,68]. RES at 45 mg/kg significantly improved osteoarthritis symptoms in mice [67], while oral administration of RES at 75 mg/kg/day did not cause apparent hepatic toxicity in mice with diet-induced obesity [68].

To determine an appropriate dose of RES for our study, mice (3 mice/group) were administered ASE (10 mg/kg body weight/day) by oral gavage in combination with RES at either 45 or 75 mg/kg/day. ASE and RES were dissolved in DMSO and subsequently mixed with corn oil to prepare the oral gavage solution, with each mouse receiving 100 µL daily. Preliminary results showed that both doses of RES effectively inhibited ASE-induced PXR activation in the liver (Supplementary Figure S3). Furthermore, our recent study demonstrated that RES at 45 mg/kg/day effectively inhibited cannabidiol-induced PXR activation in mice [59]. Thus, RES at 45 mg/kg/day was selected for subsequent experiments.

Groups of 8-week-old male C57BL/6 WT mice were orally administered ASE (10 mg/kg body weight/day) with or without the PXR-specific antagonist RES (45 mg/kg/day) by gavage for 7 days on a semisynthetic low-fat AIN76 diet containing 0.02% cholesterol (Research Diet, D00110804C). ASE and RES were dissolved in DMSO and subsequently mixed with corn oil to prepare the oral gavage solution, with each mouse receiving 100 µL of the gavage mixture daily. The AIN-76 diet has been previously used in studies of dyslipidemia and atherosclerosis in mice [9,11]. Sample size was determined based on the differences in means and standard deviation of plasma total cholesterol levels between control and dicyclohexyl phthalate treated WT mice on AIN76 diet in our recent study [9] to estimate the minimum number of animals required to significantly differentiate two groups at a *p* value of 0.05 and a power level of 90% [63,64]. The LaMorte’s Power Calculator Microsoft Excel spreadsheet was downloaded from the website [65]. On the day of euthanasia, mice (6 mice/group) were fasted for 6 h following the feeding cycle. Body weight was recorded, and mice were anesthetized with ketamine/xylazine (100/10 mg/kg) by intraperitoneal injection. The peritoneal and thoracic cavities were then opened, and blood was collected by left ventricular puncture. Following blood collection, the circulatory system was perfused with saline through the left ventricle after nicking the right atrium. Major organs were subsequently collected and weighed. Mice were housed in temperature-controlled animal facilities under a 12 h light/dark cycle. All animal procedures were performed in accordance with protocols approved by the Institutional Animal Care and Use Committee of the University of Nebraska at Kearney.

### 4.6. Plasma Analysis

Plasma total cholesterol and triglyceride concentrations were measured enzymatically using colorimetric assays according to the manufacturer’s protocol (Cholesterol 999–02601 and triglyceride 994–02891, Fujifilm, Valhalla, NY, USA). Blood samples were collected into heparin-containing tubes and centrifuged at 1500 × *g* at 4 °C for 15 min. Plasma was collected and stored at −80 °C until analysis. The LDL/VLDL and HDL lipoprotein fractions were quantified using HDL and LDL/VLDL Cholesterol Assay Kit as per the manufacturer’s instructions ( ab65390, Abcam, Waltham, MA, USA).

### 4.7. RNA Isolation and Real-Time Quantitative PCR Analysis

Total RNA was isolated from mouse tissues or cultured cells using TRIzol Reagent following the manufacture’s protocol (Thermo Fisher Scientific, 15596026). RNA concentrations and quality were assessed using NanoDrop Spectrophotometers (Thermo Fisher Scientific, ND-ONE-W). Two micrograms of total RNA were reverse-transcribed into cDNA using SuperScript III reverse transcriptase (18080093, Invitrogen, Carlsbad, CA, USA). Quantitative real-time PCR (QPCR) was performed using PowerUp SYBR Green Master Mix (Thermo Fisher Scientific, A25777) on the Applied Biosystems QuantStudio 5 Real-Time PCR System (Thermo Fisher Scientific, A34322) as per the manufacturer-supplied protocol. The gene-specific primers were purchased from Integrated DNA Technologies, and the sequences of primer sets were listed in Supplementary Table S2. For each biological sample, two technical replicate cycle threshold (Ct) values were collected and averaged. The mean Ct values were normalized to glyceraldehyde-3-phosphate dehydrogenase (GAPDH), and the relative mRNA expression levels were calculated using the comparative ΔΔCt method [69]. The relative gene expression was presented as fold change relative to the corresponding control samples.

### 4.8. Cholesterol Uptake Assay

To investigate the roles of ASE-induced PXR activation in intestinal cholesterol uptake, human intestinal LS180 cells were seeded in a black flat bottom 96 well plate and cultured to approximately 50% confluence. Cholesterol uptake was measured using Abcam Cholesterol Uptake Assay Kit following the manufacturer’s manual (Abcam, ab236212). A previous study demonstrated that treatment with 50 µM RES effectively reduced rifampicin-induced PXR activity in hPXR-overexpressing human intestinal LS174T cells without affecting cell viability [28]. In addition, our previous study showed that 50 µM RES effectively inhibited PXR signaling in human intestinal LS180 cells [59]. Therefore, we used 50 µM of RES to determine whether inhibition of PXR signaling could attenuate ASE-induced cholesterol uptake. Briefly, LS180 cells were treated with 20 µM of ASE or DMSO control in the presence or absence of 50 µM of PXR antagonism RES in serum-free culture medium containing 20 μg/mL of green fluorescent protein (GFP)-tagged cholesterol for 24 h. At the end of the treatment period, cholesterol uptake was quantified using Synergy H1 Hybrid Reader at an excitation/emission wavelength of 485/535 nm. Cholesterol uptake was expressed as fold change in relative fluorescence units (RFU) compared with the DMSO vehicle control.

### 4.9. Statistical Analysis

All data are presented as the mean ± SEM, with individual data points shown in the figures. Pairwise comparisons were analyzed by two-sample, two-tailed Student’s *t*-test. For comparisons involving multiple groups, one-way analysis of variance (ANOVA) followed by Dunnett’s *t* test was used when comparing multiple groups with a control. Two-way ANOVA followed by Bonferroni’s multiple comparisons test was used for analyses involving two independent variables. Sample sizes (n) are indicated in the figure legends. The *p* value < 0.05 was considered statistically significant. Two-way ANOVA was performed using SigmaPlot 13.0, while all other statistical analyses were conducted using GraphPad Prism.

## 5. Conclusions

A non-phthalate plasticizer ASE is linked to the pro-atherosclerotic PXR signaling pathway for the first time and is established as a potent and selective PXR agonist. The key amino acid residues Trp299, His407, Arg410, Phe420, and Phe429 were identified within the human PXR ligand-binding pocket that interacts with ASE. The in vivo study in wildtype C57BL/6 mice suggests ASE feeding increases plasma total cholesterol and atherogenic LDL cholesterol levels in mice consistent with PXR involvement. Mechanistically, ASE induces cholesterol uptake by intestinal cells mediated by PXR. Our study will contribute to a better understanding of how gene–environment interactions may predispose individuals to cardiovascular disease.

## Data Availability

The original contributions presented in this study are included in the article/Supplementary Materials. Further inquiries can be directed to the corresponding author.

## Supplementary Materials

The following supporting information can be downloaded at https://www.mdpi.com/article/10.3390/ijms27188189/s1.

## Abbreviations

The following abbreviations are used in this manuscript:

ASE	Alkylsulfonic phenyl ester
PXR	Pregnane X receptor
CVD	Cardiovascular disease
EDC	Endocrine disrupting chemical
PVC	Polyvinyl chloride
DEHP	Di(2-ethylhexyl) phthalate
RES	Resveratrol
MTT	3-(4,5-dimethylthiazol-2-yl)-2,5-diphenyltetrazolium bromide
RAR	Retinoid acid receptor
RXR	Retinoid X receptor
FXR	Farnesoid X receptor
LXR	Liver X receptor
PPAR	Peroxisome proliferator-activated receptor
VDR	Vitamin D receptor
CAR	Constitutive androstane receptor
ER	Estrogen receptor
SMRT	Silencing mediator of retinoid and thyroid hormone
NCoR	Nuclear receptor co-repressor
MD	Molecular dynamics
LBD	Ligand-binding domain
DBD	DNA binding domain
WT	wildtype
BW	Bodyweight
LDL	Low-density lipoprotein
VLDL	Very low-density lipoprotein
HDL	High-density lipoprotein
CYP	Cytochrome P450
MDR	Multidrug resistance
GST	Glutathione transferase
NPC1L1	Niemann–Pick C1-Like 1
MTP	Microsomal triglyceride transfer protein
NR	Nuclear receptor
TBC	Tributyl citrate
ApoE	Apolipoprotein E
β-gal	β-galactosidase
DMSO	Dimethyl sulfoxide
RLU	Relative Light Unit
EC_50_	Half-maximal effective concentration
GAPDH	Glyceraldehyde-3-phosphate dehydrogenase
ANOVA	Analysis of variance
