# Supplementary material for: A Universal Plasticizer Alkylsulfonic Phenyl Ester Activates Pregnane X Receptor to Increase Intestinal Cholesterol Uptake
Source: Int J Mol Sci. 2026 Sep 15;27(18):8189. doi: 10.3390/ijms27188189 (PMC13607809; doi:10.3390/ijms27188189)
Supplement: Supplementary file 1 [file ijms-27-08189-s001.zip › ijms-4534374-supplementary.pdf]

Supplementary Materials

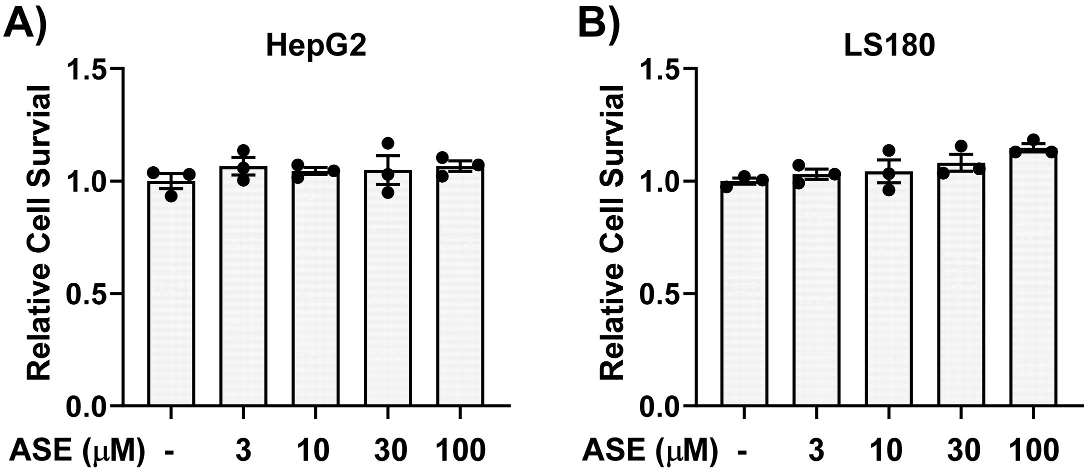

Figure S1. ASE does not affect cell viability.
HepG2 cells (A) and LS180 cells (B) were treated with ASE at the indicated concentrations for 24 hours, followed by MTT assay (n=3). Relative cell survival was calculated relative to vehicle DMSO controls. No significant change in cell viability was observed at any concentration tested compared with DMSO control.

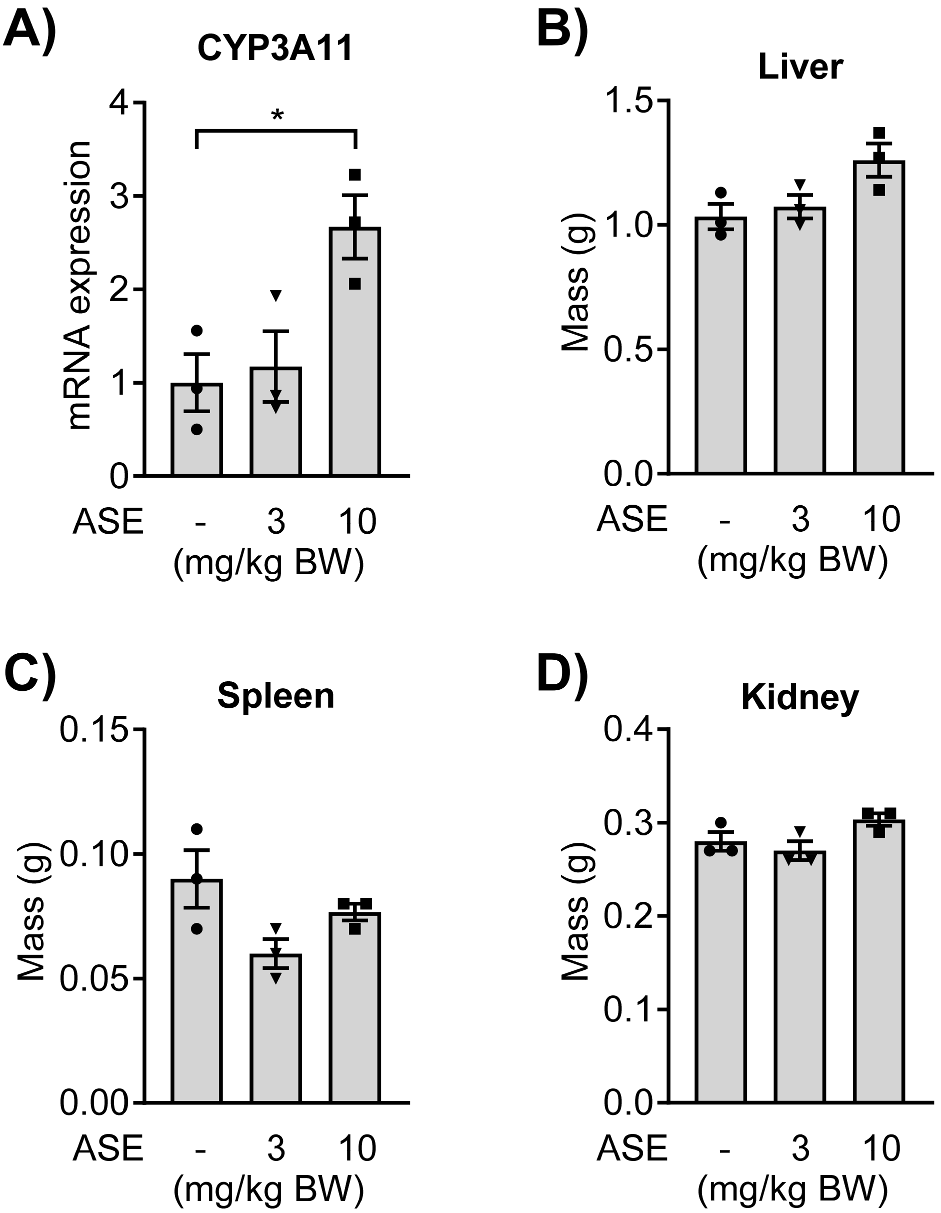

Figure

Figure S2. The effects of ASE exposure on hepatic PXR target gene expression and organ weight in WT mice.

Male WT mice at the age of 8 weeks old were treated with vehicle control or ASE (3 or 10 mg/kg/day) by oral gavage for 7 days on chow diet (3 mice/group). **(A)** Hepatic expression of PXR target gene *CYP3A11* was measured by QPCR (n=3, one-way ANOVA, Dunnett's t test for multiple comparisons to control, *P<0.05). **(B-D)** The major organs were weighed at anesthesia, including liver, spleen, and kidney. Error bars represent ± SEM.

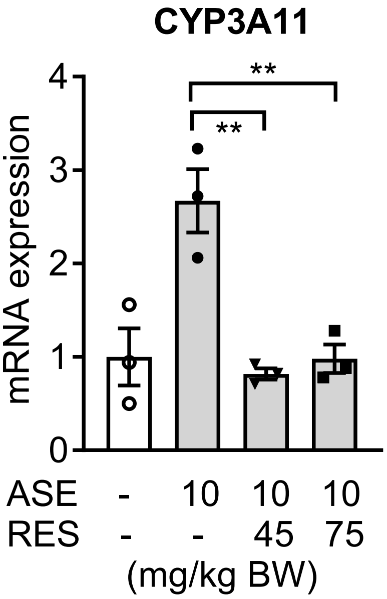

Figure S3. The impacts of RES on ASE-induced mRNA expression of hepatic PXR target gene *CYP3A11* in WT mice.

Eight-week-old male WT mice were treated with vehicle control or ASE (10 mg/kg/day) with/without PXR specific antagonist RES (45 or 75mg/kg/day) by oral gavage for 1 week on chow diet (3 mice/group). Hepatic expression of PXR target gene *CYP3A11* was measured by QPCR (n=3, one-way ANOVA, Dunnett's t test for multiple comparisons to ASE (10 mg/kg/day) group, **P<0.01). Error bars represent ± SEM.

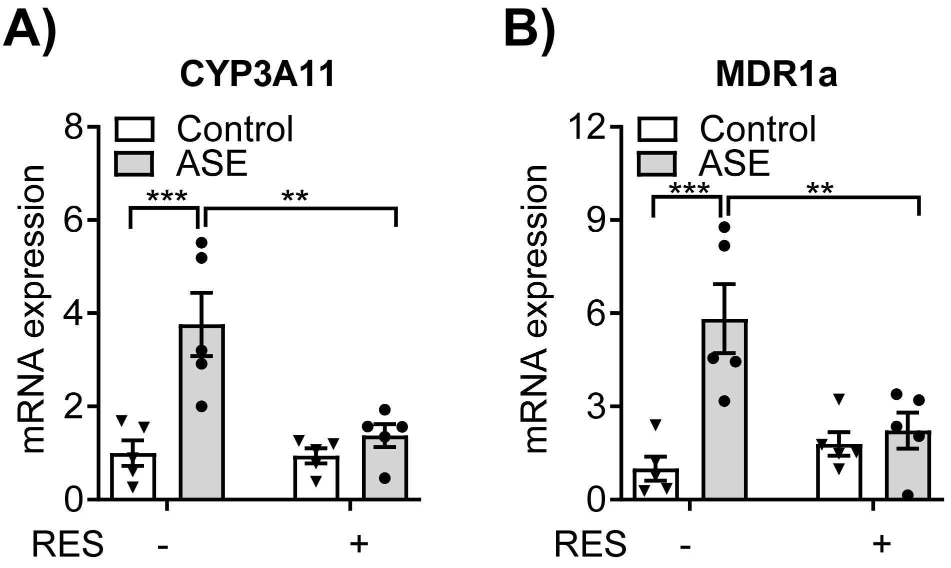

Figure S4. The influences of ASE exposure on hepatic PXR target genes in WT mice. Male WT mice at the age of 8 weeks old were administrated with ASE (10 mg/kg/day) and/or RES (45 mg/kg/day) or vehicle control by oral gavage for 7 days on AIN76 diet. Hepatic expression of PXR target genes, *CYP3A11* (A) and *MDR1a* (B), was measured by QPCR (n=5, two-way ANOVA, Bonferroni multiple comparisons test for multiple comparisons, $^{**}P<0.01$ and $^{***}P<0.001$). Error bars represent ± SEM.

Table S1. Primer sequences for site-directed mutagenesis

Mutant	Primer sequences
PXR- F281L	5'-CCTGCTGAAGGGGGCCGCTTTGGAGCTGTGTCAACTGAG-3'
	5'-CTCAGTTGACACAGCTCCAAAGCGGCCCCCTTCAGCAGG-3'
PXR-F288A	5'-CGAGCTGTGTCAACTGAGAGCCAACACAGTGTTCAACGCGG-3'
	5'-CCGCGTTGAACACTGTGTTGGCTCTCAGTTGACACAGCTCG-3'
PXR-W299L	5'-CAACGCGGAGACTGGAACCTTGGAGTGTGGCCGGCTGTCC-3'
	5'-GGACAGCCGGCCACACTCCAAGGTTCCAGTCTCCGCGTTG-3'
PXR-H407A	5'-CCGCAGCATCAATGCTCAGGCCACCCAGCGGCTGCTGCGCATCC-3'
	5'-GGATGCGCAGCAGCCGCTGGGTGGCCTGAGCATTGATGCTGCGG-3'
PXR-R410L	5'-GCATCAATGCTCAGCACACCCAGCTGCTGCTGCGCATCCAGG-3'
	5'-CCTGGATGCGCAGCAGCAGCTGGGTGTGCTGAGCATTGATGC-3'
PXR-F420L	5'-CGCATCCAGGACATACACCCCTTAGCTACGCCCCTCATGCAGG-3'
	5'-CCTGCATGAGGGGCGTAGCTAAGGGGTGTATGTCCTGGATGCG-3'
PXR-F429L	5'-CCCTCATGCAGGAGTTGTTGGGCATCACAGGTAGC-3'
	5'-GCTACCTGTGATGCCCAACAACTCCTGCATGAGGG-3'

Table S2. Primer sequences for QPCR

Genes	Primer sequences
Mouse CYP3A11	5’-CAGCTTGGTGCTCCTCTACC-3’
	5’-TCAAACAACCCCCATGTTTT-3’
Mouse MDR1a	5’-CCCCCGAGATTGACAGCTAC-3’
	5’- ACTCCACTAAATTGCACATTTCCTTC-3’
Mouse NPC1L1	5’-TTGCCTTGACCTCTGGCTTAG-3’
	5’-AGGGCGGATGAATCTGTGC-3’
Mouse MTP	5’-TGAGCGGCTATACAAGCTCAC-3’
	5’-CTGGAAGATGCTCTTCTCGC-3’
Mouse GAPDH	5’-AACTTTGGCATTGTGGAAGG-3’
	5’-GGATGCAGGGATGATGTTCT-3’
Human CYP3A4	5’-GGCTTCATCCAATGGACTGCATAAAT-3’
	5’-TCCCAAGTATAACACTCTACACAGACAA-3’
Human MDR1	5’-CCCATCATTGCAATAGCAGG-3’
	5’-GAGCATACATATGTTCAAACTTC-3’
Human NPC1L1	5’-CTTCTACCAGCATAGCTTTGCC-3’
	5’-AGAGCCATACACGCCACAC-3’
Human MTP	5’-ACAAGCTCACGTACTCCACTG-3’
	5’-TCCTCCATAGTAAGGCCACATC-3’
Human GAPDH	5’-GGCCTCCAAGGAGTAAGACC-3’
	5’-AGGGGAGATTCAGTGTGGTG-3’
